# Better score function for peptide identification with ETD MS/MS spectra

**DOI:** 10.1186/1471-2105-11-S1-S4

**Published:** 2010-01-18

**Authors:** Xiaowen Liu, Baozhen Shan, Lei Xin, Bin Ma

**Affiliations:** 1 David R. Cheriton School of Computer Science, University of Waterloo, Canada; 2 Bioinformatics Solutions, Inc., Waterloo, Canada; 3 Department of Computer Science, University of Western Ontario, Canada

## Abstract

**Background:**

Tandem mass spectrometry (MS/MS) has become the primary way for protein identification in proteomics. A good score function for measuring the match quality between a peptide and an MS/MS spectrum is instrumental for the protein identification. Traditionally the to-be-measured peptides are fragmented with the collision induced dissociation (CID) method. More recently, the electron transfer dissociation (ETD) method was introduced and has proven to produce better fragment ion ladders for larger and more basic peptides. However, the existing software programs that analyze ETD MS/MS data are not as advanced as they are for CID.

**Results:**

To take full advantage of ETD data, in this paper we develop a new score function to evaluate the match between a peptide and an ETD MS/MS spectrum. Experiments on real data demonstrated that this newly developed score function significantly improved the *de novo *sequencing accuracy of the PEAKS software on ETD data.

**Conclusion:**

A new and better score function for ETD MS/MS peptide identification was developed. The method used to develop our ETD score function can be easily reused to train new score functions for other types of MS/MS data.

## Background

In recent years, tandem mass spectrometry (MS/MS) has become a popular technique in proteomics for protein identification. In a typical "bottom-up" approach, proteins are enzymatically digested into short peptides, each of them is measured with MS/MS and produces an MS/MS spectrum. Analytical software is used to identify a peptide for each qualified spectrum. Then the peptides are used for protein identification and characterization. Many software programs (e.g. Mascot, Sequest and PEAKS) have been developed for MS/MS data analysis [1-6].

Database search and *de novo *sequencing are two main approaches for peptide identification from MS/MS data. Database search requires a protein sequence database, and tries to find a peptide from the database that best explains the MS/MS spectrum. *De novo *sequencing does not require a sequence database, and instead constructs a peptide sequence from scratch to best explain the MS/MS spectrum. Algorithms and software developments using these two approaches have been one of the research focuses in bioinformatics community recently. Some exemplary work for database search approach can be found in [1,2,7-10], and for *de novo *sequencing approach can be found in [3,11-18].

**Figure 1:**
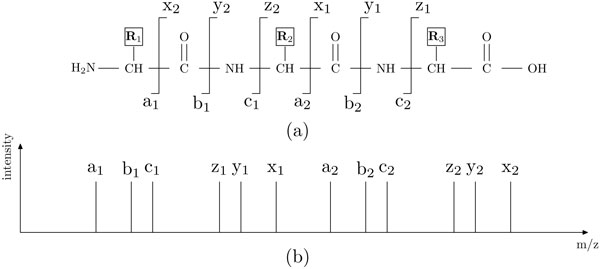
**Peptide fragmentation**. (a) For every two adjacent amino acid residues, there are three positions where the peptide backbone can be fragmented, resulting in six types of ions. (b) Each ion type may generate a peak at the corresponding m/z value in the theoretical spectrum.

**Figure 2:**
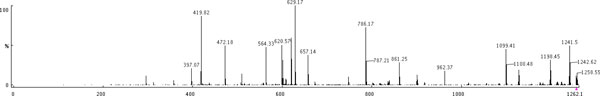
**A real tandem mass spectrum**.

Both approaches identify peptides with two steps. First, a set of candidate peptides are selected either from a protein database or with a sequence construction algorithm for a given spectrum. Second, each sequence candidate is evaluated with a score function that measures the quality of the match between the sequence and the spectrum, and the peptide with the best matching score is then output. Inevitably, the quality of the score function greatly affects the quality of the peptide identification and its following analysis. In a tandem mass spectrometer, a peptide ion is fragmented into fragment ions. Normally, the fragmentation can occur at any position of the peptide backbone, resulting in different types of fragment ions (Figure 1(a)). Each ion will result in a peak at the corresponding mass to charge ratio (m/z), and all the ions together form a theoretical mass spectrum (Figure 1(b)). The real MS/MS spectrum is more complicated due to the existence of noise peaks, the missing of some fragment ion peaks, and the different intensities of each peak (Figure 2).

The collision induced dissociation (CID) is among the earliest ways developed for peptide fragmentation in a tandem mass spectrometer, and is also the most widely studied in bioinformatics. It is known to produce more y and b-ions than the other ion types. Recently, more fragmentation mechanisms such as infrared multiphoton dissociation (IRMPD) [19], electron capture dissociation (ECD) [20], and electron transfer dissociation (ETD) [21] have been developed. Particularly, ETD is regarded as a promising method complementary to CID because ETD is better suited for sequencing larger and more basic peptides.

Due to the importance of the matching score function, many scoring methods have been proposed. One straightforward approach is based on the match between the real spectrum and the theoretical spectrum generated from a candidate peptide [2,22]. Another approach is based on the probabilistic model of fragmentation process [9,12,23,24]. These probabilistic models study and employ the statistical differences between peaks produced with different ion types. Frank *et al*. further exploited these properties by a probabilistic network for collision induced dissociation (CID) data to improve the accuracy of *de novo *sequencing [13].

Most of the existing score functions are designed and trained specifically for CID data. ETD spectra differ from CID spectra in the magnitude and the complexity of fragment ion signals. Although a few software tools such as PEAKS and Mascot have been adjusted to work on ETD data, they are better suited for CID data and give poorer results with ETD data. To better utilize the advantage of ETD spectra on longer and more basic peptides, in this paper, we propose a score function for ETD data based on a probabilistic model. The experiments on real data showed that the score function significantly improved the *de novo *sequencing performance when used in the PEAKS software [3]. Our method for developing this score function is general enough to be used to develop new score functions for CID and other types of MS/MS data.

## Results

We used an ETD dataset of 9885 spectra from a complex *C. elegans *protein mixture digested with trypsin followed by alkylation with iodoacetamide. The spectra were acquired on an LTQ Orbitrap XL ETD. The peptide mixtures were separated with Surveyor LC equipped with MicroAS autosampler using a reversed phase peptide trap and a reversed phase analytical column at a flow rate of 250 nl/min. A gradient of 5 ~ 30% acetonitrile in 90 minutes was employed.

The database search modules in PEAKS 5.0 [3] and Mascot 2.2 [1] were used for peptide identification. The tandem mass spectra were matched against NCBI *C. elegans *protein sequence database. For both programs, the error tolerances for parent mass and fragment mass were set as 10 ppm and 0.8 Da, respectively. Each program reported no peptide or one best peptide with a confidence score for each spectrum. (The programs may report no peptide for low quality spectra.) PEAKS 5.0 reported 1496 peptides and Mascot 2.2 reported 2950 peptides. We applied several filters to remove low quality spectra. A spectrum is kept only if it satisfies the following three conditions: (1) the PEAKS confidence score is no less than 50%; (2) the Mascot confidence score is no less than 30%; and (3) the two peptides reported by PEAKS and Mascot are the same. Finally, we selected 259 spectrum-peptide pairs from the dataset. Both programs identified the peptides consistently with high confidence scores. Thus, the peptides are very accurate and can be used as positive controls in our experiments. Among them, 130 spectrum-peptide pairs are used for training and the remaining 129 pairs are used for testing.

An ETD spectrum often contains several peaks generated from its precursor ions. These peaks often have high intensities, and may affect the performance of the matching score function. Thus, we removed these precursor ion peaks from the spectra in data preparation.

Most *de novo *sequencing software developed so far is for CID data and does not work for ETD data. The only software available to us for performance comparison using ETD data is the PEAKS software [3]. Recent versions of PEAKS have a built-in set of parameters for ETD, which was adjusted from its CID parameters. We compared our score function with the function used in PEAKS 5.1. To make a fair comparison, the result of our new score function is obtained by simply replacing the score function of PEAKS 5.1 without making any change to the algorithm. Thus, the improvements reported in the following are purely caused by the difference in the score functions.

We mainly compared the accuracies of the PEAKS *de novo *sequencing results with the two score functions, respectively. There are two ways to define the accuracy:

and

Accuracy II is the same as the accuracy defined in [13]. We note that Accuracy II is a fair comparison measure only if two methods output similar number of predicted amino acids. Otherwise, the accuracy of the method with shorter output will be unfairly boosted. For this reason, we also show the average prediction length of the two methods in Table 1.

**Table 1:** Comparison of the score function in PEAKS and the new score function.

Score function	Accuracy I	Accuracy II	Average length	Predictions with correct subsequences of length at least *x*							
				
				*x *= 3	4	5	6	7	8	9	10
PEAKS 5.1	0.325	0.342	13.70	0.56	0.46	0.40	0.31	0.24	0.16	0.13	0.12
New score	0.553	0.559	14.26	0.84	0.77	0.64	0.54	0.43	0.37	0.32	0.28

In addition, we search the maximum correct consecutive subsequences in the predicted peptides. For each predicted peptide *P*, the length of the maximum correct consecutive subsequence is denoted by *L*_*max*_(*P*). We count the number of predicted peptides with *L*_*max*_(*P*) ≥ *l*, for *l *= 3, 4, ..., 10. The error tolerance for the position of a predicted amino acid subsequence is 0.6 Da, and the amino acids leucine(L) and isoleucine(I) are considered as the same since their mass values are the same.

The testing data contain 129 peptides and 1859 amino acids. Using the function in PEAKS 5.1 and our score function, we acquired two sets of predicted peptides, each contains 129 *de novo *peptides. The accuracy and the maximum correct consecutive subsequences of the resulting peptides are reported in Table 1. The results show that the new score function significantly improves the accuracy of *de novo *sequencing from 32.5% to 55.3% for Accuracy I, and from 34.2% to 55.9% for Accuracy II. In PEAKS 5.1, about 56% of predicted peptides contain a corrected subsequence with length at least 3. For the new score function, more than half of the predicated peptides contain a correct subsequence with length at least 6. From the experiments, we conclude that the new score function has better performance in *de novo *sequencing with ETD data than the score function in PEAKS 5.1.

## Discussion

We note that the independence assumption in our score function development in Section Methods is not very realistic because different types of ions generated by the fragmentation between the same pair of adjacent residues of the peptide are often correlated to each other. To more accurately model this, a probabilistic network (or Bayesian network) can be used similarly to [13]. We also tried this approach on our data and found no apparent improvement over the model used in this paper. One possible reason is that our training data is of limited size, and not sufficient to train the many parameters of the probabilistic network accurately. Another possible reason is the following. The probabilistic network is advantageous over our simple model only in dealing with the dependence between different types of fragment ions at the same location (between the same pair of adjacent residues). However, when a *de novo *sequencing method makes an error at a location, normally there is either no fragment ion or only one low fragment ion at the location. Under this condition, the dependence between different types of fragment ions is weak. As a result, a model with the independence assumption would work as well as a more sophisticated one without this assumption.

## Conclusion

In this paper, we proposed a significance level measurement to distinguish signal peaks from noise. Based on this, a score function for *de novo *sequencing with ETD data was defined. Experiments on real data showed that our score function greatly improved the score function used in the latest PEAKS 5.1 software. The method used to develop our ETD score function can be easily reused to train new score functions for other types of MS/MS data.

## Methods

We first introduce a novel *significance level *measurement to evaluate the likelihood that a peak is signal. The significance level has a better ability to distinguish signal and noise peaks than the original relative intensities given by the spectrum. Then, we study the distributions of the significance levels of different types of fragment ions, and define a likelihood ratio score for each fragment ion. Finally, we combine all the fragment ion scores of a peptide together to provide a peptide score function.

### The peak significance level

The peptide fragmentation mechanism shown in Figure 1(a) and 1(b) is overly simplified. In a real mass spectrum (Figure 2), there are also many noise peaks that cannot be explained by the six types of fragment ions. Meanwhile, not all of these fragment ions form strong peaks in the spectrum. Some are either absent or too low to be distinguished from the noise. Different spectra, and even different regions of the same spectrum, may have different noise and signal levels. Consequently, the intensity or the relative intensity given by the original spectrum do not accurately reflect the likelihood for a peak being signal. Thus, it is necessary to develop a better measure to reflect this likelihood. In this section we focus on this task and develop a measure called *significance level*.

According to our experience, four features of a peak may be associated with the significance of the peak: (1) global rank, (2) local rank, (3) global intensity ratio, and (4) local intensity ratio. These four features are explained in the following, respectively.

The global rank of a peak is the number of peaks (in the same spectrum) that are higher than or equal to the current peak. A smaller global rank means a more significant peak. However, a spectrum of a longer peptide normally has more signal peaks than a spectrum of a shorter peptide. With this observation, we further adjust the global rank by dividing the original ranking number by the size of the peptide, which is estimated by dividing the precursor ion mass by the average mass of an amino acid residue (approximately 112 Da).

**Figure 3:**
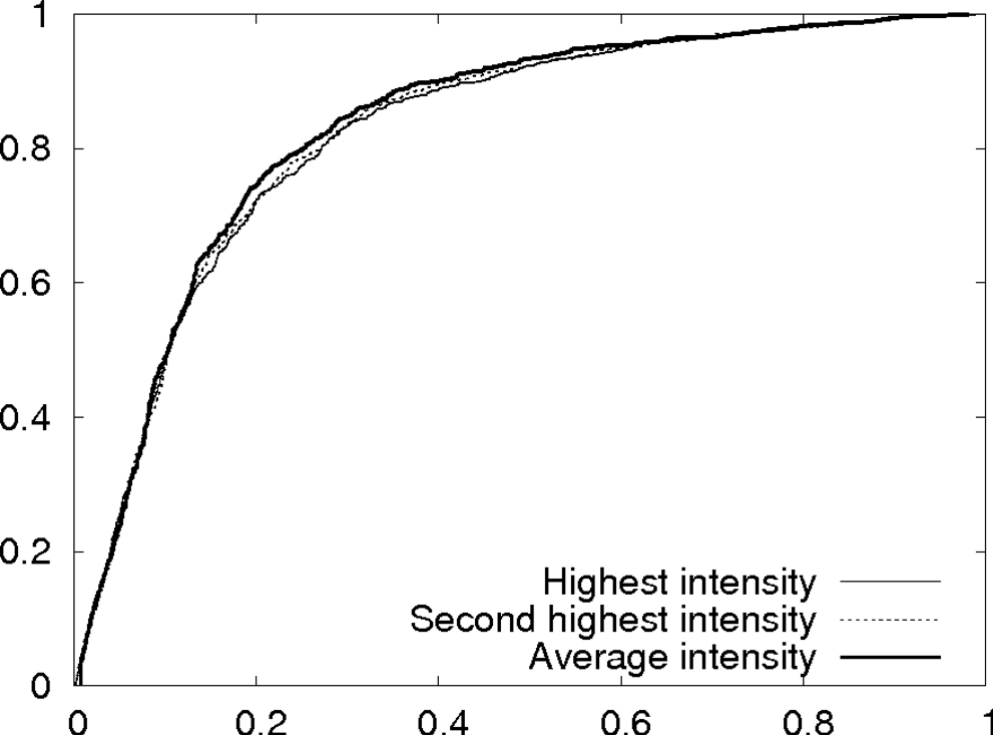
**Comparison of the three reference intensities**. The ROC curves for the three reference intensities when the global intensity ratio is used to distinguish z' signal peaks and background noise peaks.

The global intensity ratio is the ratio between a *global reference intensity *and the intensity of the considered peak. If this ratio is lower than 1 then it is set to 1. We consider the following three choices for calculating the global reference intensity: (a) the intensity of the highest peak, (b) the intensity of the second highest peak, and (c) the average intensity of the 3rd to the 10th highest peaks. The reason we consider choices (b) and (c) is that some peptides are hard to fragment or can only fragment on one or two sites, resulting in a spectrum with only a couple of very high intensity peaks and many very low signal peaks. The very few extremely high intensity peaks are really outliers and cannot serve as an accurate reference. Therefore, it is meaningful to use choices (b) or (c), which "boost" the low-intensity signal peaks. The three choices are compared using the training data of 130 spectrum-peptide pairs described in Section Results as follows: We consider z'-ions, which are z-ions with an additional hydrogen. There are in total 1206 z'-ions for the peptides in the training data, of which 1093 match peaks. These 1093 peaks are regarded as signal peaks. Then 12060 (10 times of the real z'-ions) random m/z values are generated to match peaks in the same set of spectra, giving 3370 randomly matched peaks. These 3370 random peaks are regarded as background noise. For each of the three choices of the global reference intensity, we draw the Receiver Operating Characteristic (ROC) curve for distinguishing the signal peaks from the background noise. Figure 3 shows the three curves. Choice (c) has the best ROC curve and is therefore used in this paper to calculate the global reference intensity and the global intensity ratio.

The local rank and the local intensity ratio are defined similarly as their global versions, except that only the peaks within ±57 Da (the mass of the smallest amino acid residue) difference to the current peak are examined. More specifically, the local rank is the number of peaks which are within ±57 Da difference, and are higher than or equal to the current peak. Suppose the intensity of the highest peak within ±57 Da is *h*_1_, the global reference intensity is *h*_2_, the intensity of the current peak is *h*, then the local intensity ratio of the current peak is defined as max (1, min(*h*_1_, *h*_2_)/*h*).

All of the above four features contribute some information towards the significance of a peak. The significance level of a peak is calculated as a linear combination of the natural logarithms of the four features. Let *r*_*g*_, *r*_*l*_, *t*_*g *_and *t*_*l *_be the global rank, local rank, global intensity ratio and local intensity ratio of a peak, then its significance level is

where *c*_*rg*_, *c*_*rl*_, *c*_*tg *_and *c*_*tl *_are the coefficients for the features. It should be noted that under this definition, a smaller value for significance level means a stronger peak. This is somewhat counterintuitive but chosen deliberately in order to assign the same value 0 to the strongest peak in every spectrum.

**Figure 4:**
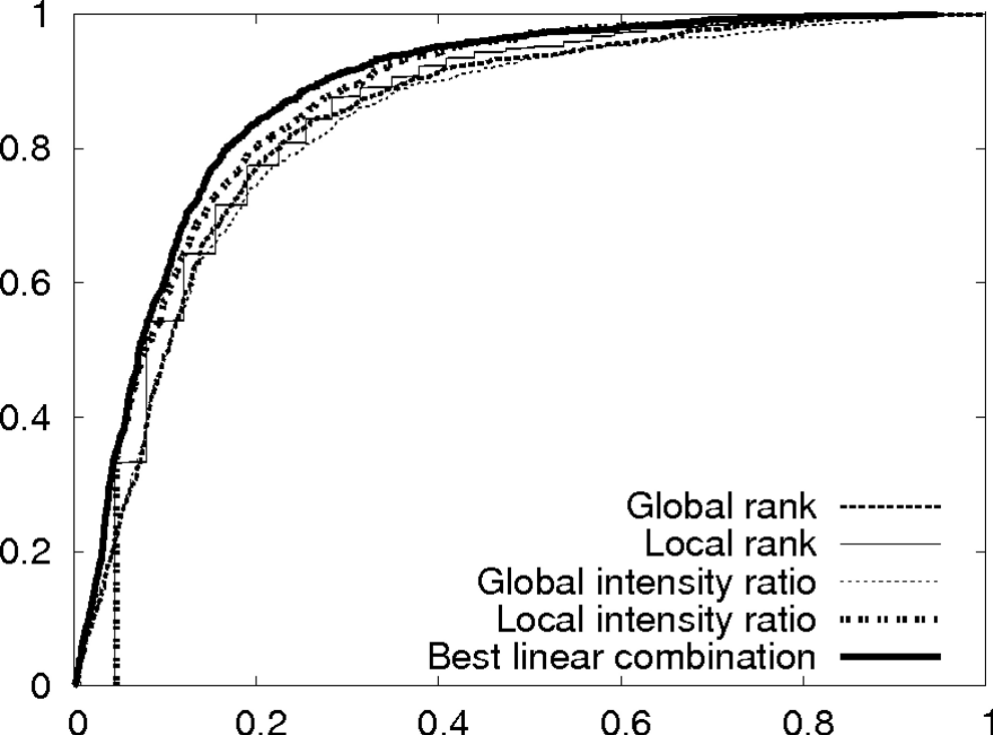
**The ROC curves for the four features and their best linear combination**.

From our experience, the local rank is the most important feature. An exhaustive search is used to find the best coefficient combination. We let *c*_*rl *_= 1, and enumerate all possible values of *c*_*rg*_, *c*_*tg*_, *c*_*tl *_∈ {0.01, 0.02, ..., 1}. For each combination, the significance level is evaluated by the area below the ROC curve for distinguishing the signal peaks and the background noise in the training data. The search found the best normalized coefficients to be *c*_*rg *_= 0.22, *c*_*rl *_= 0.40, *c*_*tg *_= 0.05 and *c*_*tl *_= 0.33. The ROC curves with each of the four features and their best combination for the testing data are shown in Figure 4. This figure illustrates that the linear combination of the four features noticeably improves the ability in distinguishing between real matched peaks and randomly matched peaks. We point out that for *de novo *sequencing, the differences between the top few sequence candidates are minor, therefore a small improvement in Figure 4 may cause a large improvement in the final *de novo *sequencing results.

### Distribution of the peak significance levels for different ion types

It is well known that different types of mass spectrometers (especially if the peptide fragmentation methods are different) tend to produce different ion types. For example, CID produces more b and y-ions than other ion types, while ETD produces more c and z'-ions. Thus, when using a peptide to explain a spectrum, matching a high-intensity peak with a z'-ion or a y-ion will have different contributions towards the likelihood that the peptide is correct. For ETD data, because we expect high z'-ions, the matching with a z'-ion will contribute more than the matching with a y-ion. Thus it is necessary to study the distribution of the significance level for different ion types. In this section we study this with ETD data. The distribution will help us to develop our final score function.

**Figure 5:**
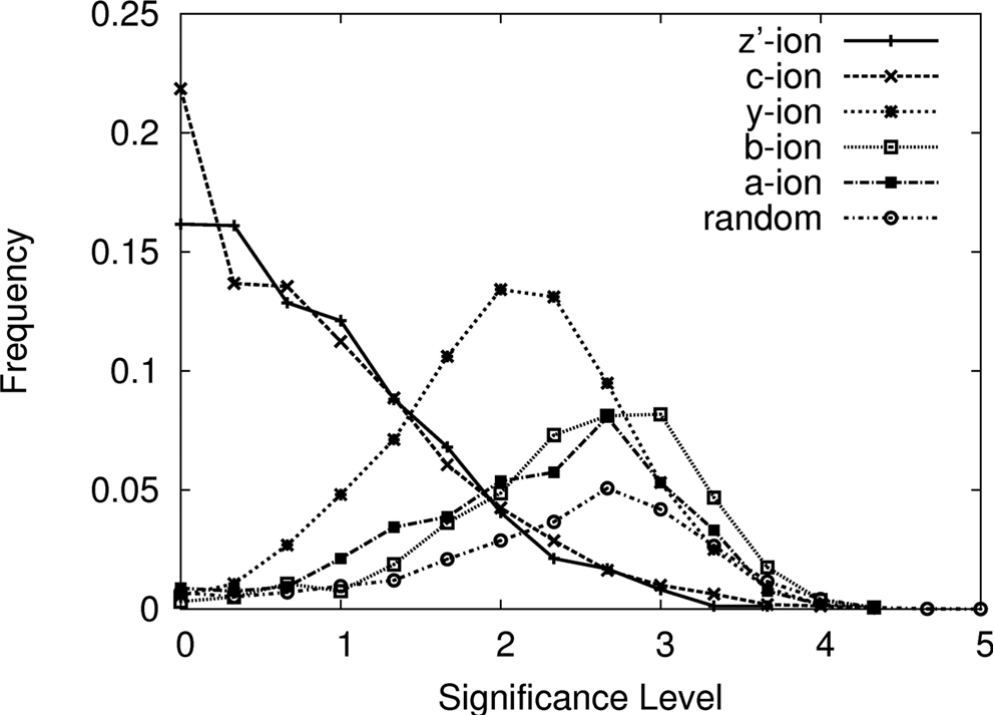
**The distributions of the significance levels for different ion types and random peaks**.

By using the same training data of 130 spectrum-peptide pairs, the distribution of the significance levels for several common ion types in ETD data are plotted in Figure 5. Keep in mind that the significance level 0 means the peak is the most significant in a spectrum.

Because the training data is limited in its size (only approximately 1000 data points for each ion type), the distributions shown in Figure 5 are not smooth and not suitable to be used directly in our scoring function. Thus the following treatment is applied to the data. For each ion type *t*, suppose there are *n *ions of this type from the given peptides. *m *of the *n *ions match peaks in the spectra. We divide the range of significance level into four intervals *I*_1_, *I*_2_, *I*_3_, *I*_4_, such that *I*_*j *_is left to *I*_*j*+1 _and each interval contains *m*/4 matched peaks. Thus for each *j *= 1, 2, 3, 4, *Pr*(*t *ion significance level ∈ *I*_*j*_) = . Meanwhile we can calculate *Pr*(random ion significance level ∈ *I*_*j*_) by simple counting. So, the likelihood ratio of the two events is calculated as

Next we define a continuous function *f *by interpolation to measure the significance of the event that a type-*t *peak has significance level *x*. Denote the centroid of each interval *I*_*j *_by *c*_*j*_. Define

In addition, let *c*_5 _be the centroid of the largest 10% significance levels in the training data. At significance level greater than or equal to *c*_5_, most peaks are noise. Therefore, matching one of these peaks with an ion becomes a very insignificant event, and can be treated as if the ion does not match any peak. Thus, we define

**Figure 6:**
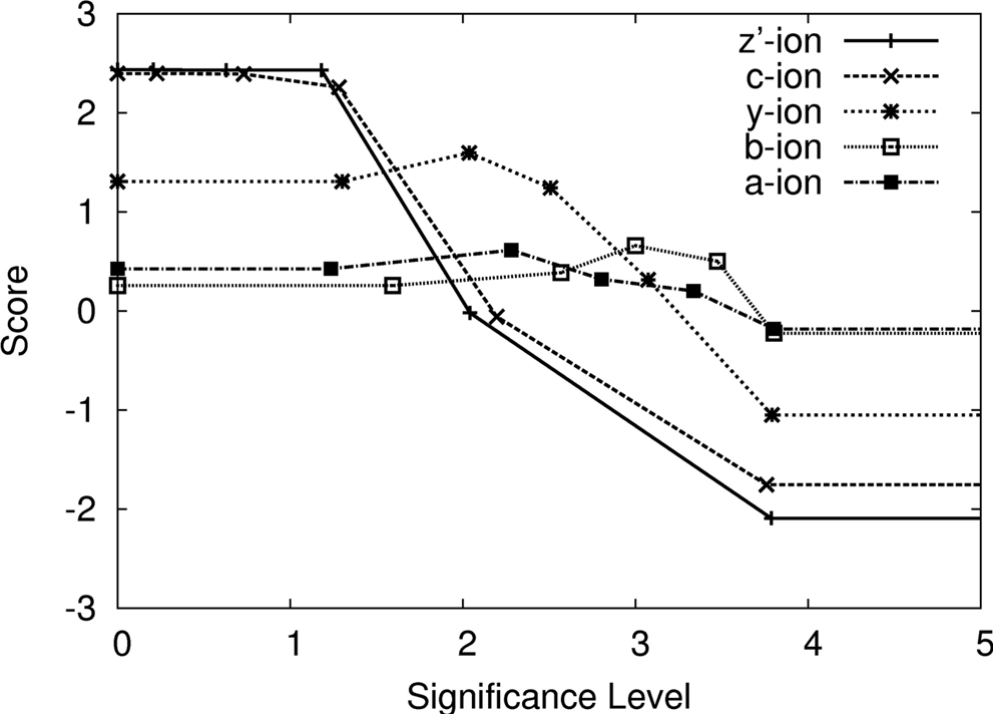
**The score functions attained from the training data for different ion types**.

Let *f*(*x*) = *f*(*c*_1_) for *x *<*c*_1 _and *f *(*x*) = *f *(*c*_5_) for *x *>*c*_5_. Let *f *(*x*) for other *x *be defined by linear interpolation. Thus, *f*(*x*) becomes an approximation to the log likelihood-ratio between the two hypotheses that the peak is a *t*-ion match and a random match. More specifically,

The curves of *f*(*x*) for several different ion types are shown in Figure 6. This function will be used to develop our final score function.

### The probabilistic model and score function

Almost every score function for measuring the peptide-spectrum matching relies on the peaks matched by the theoretical fragment ions of the peptide. In general the more significant peaks are matched, the more likely this peptide is the correct peptide. However, because there are different types of ions involved, it is not trivial to balance the contributions of all ions and combine them together as a single score value. Here we use the standard likelihood ratio method to define the score. Consider a peptide *P *of length *n*. Suppose there are *k *ion types *t*_1_, ..., *t*_*k *_considered in the score function. Denote all ions of type *t*_*i *_by *t*_*i*, *j *_(*j *= 1, ..., *n *- 1). Let *x*_*i*, *j *_be the significance level of the peak that matches *t*_*i*, *j*_. *x*_*i*, *j *_= ∞ if the ion matches no peak. Then the likelihood of observing the matches *x*_*i*, *j *_(*i *= 1, ..., *k*, *j *= 1, ..., *n *- 1) under the hypothesis that *P *is the correct peptide is

The likelihood of the matches under the hypothesis that *P *is a random peptide is

The significance of this match, denoted as *λ *(*x*_1,1_, *x*_1,2_, ..., *x*_*k*, *n*-1_), is therefore evaluated by the ratio between these two likelihoods. In this section we make the assumption that the events that *t*_*i*__, *j *_has intensity *x*_*i*__, *j *_are independent to each other. The independence assumption makes our score function easier to calculate while not sacrificing much performance on analysis accuracy. A discussion on the model where the events are dependent can be found in Section Discussion.

When the events *t*_*i*, *j *_has intensity *x*_*i*, *j *_are independent to each other, the log-likelihood-ratio becomes(1)

Here *f*_*i*, *j *_is one of the *f *functions trained for the *j*-th ion of the ion type *t*_*i*_. In practice, for each ion type of interest, we learn five *f *functions, for the first and second ions near the N-terminus, the last and second last ions at the C-terminus, and the middle of the peptide, respectively. The log *λ *defined in (1) is our score function.

We still need to decide what types of ions can be included in the score function for ETD data. Clearly we want to include the more frequently observed ion types because they are more distinguishable from noise. Meanwhile, we do not want to include ion types that are too dependent to each other because that conflicts our independence assumption. The frequencies of observing peaks for a particular ion type in our training data are listed in Table 2. From the table, we select eight ions with the highest frequencies for our score function: z', c, y, b, a, y-H_2_O, c-H_2_O, and z'^2+^-ion, where z'^2+^-ion is the z'-ion with charge 2. z-ion is not included because it is highly correlated with z'-ion.

**Table 2:** The frequencies that different fragment ion types have observed peaks in the training ETD data.

Ion	z'	c	y	z	b	a
Frequency	81.9%	79.9%	70.2%	45.3%	40.5%	37.9%

Ion	z'^2+^	y-H_2_O	C-H_2_O	z'-NH_3_	a-_NH3_	b-H_2_O

Frequency	34.3%	30.8%	29.3%	27.2%	24.9%	24.5%

## Competing interests

The authors declare that they have no competing interests.

## Authors' contributions

XL and BM designed and tested the score function, and drafted the manuscript. BS and LX contributed to the dataset and the test on the dataset.
